# The short-run effects of knowledge intensive greenfield FDI on new domestic entry

**DOI:** 10.1007/s10961-017-9575-y

**Published:** 2017-04-04

**Authors:** Sara Amoroso, Bettina Müller

**Affiliations:** 1 Joint Research Centre (JRC), European Commission, Calle Inca Garcilaso 3, 41092 Seville, Spain; 2 Centre for European Economic Research (ZEW), L 7, 1, 68161 Mannheim, Germany

**Keywords:** Foreign direct investments, Knowledge spillovers, New firm entry, L26, F21, O30

## Abstract

Existing evidence on the impact of foreign direct investment on domestic economies remains ambiguous. Positive technology spillovers of foreign investment may be outweighed by negative crowding out effect due to increased competition. In this paper, we employ a unique country/sector-level data set to investigate the impact of what is considered the ‘best’ type of foreign investment—greenfield knowledge intensive FDI—on domestic entry. Our results suggest that, in the short run, this type of FDI is positively related to the entry rate in the host country, if the domestic sector is either dynamic, or highly R&D intensive. These sectors may be respectively characterized by lower entry costs, which encourage a ‘trial-and-error’ learning business approach, and by a higher level of absorptive capacity which increases the chance of technology transfer.

**Electronic supplementary material:**

The online version of this article (doi:10.1007/s10961-017-9575-y) contains supplementary material, which is available to authorized users.

## Introduction

Foreign direct investment (FDI) is expected to transfer knowledge and technology, enhance productivity, competitiveness, and ultimately boost long-run growth of the domestic economy. Many studies have investigated what factors and government strategies attract such type of investment (Guimón 2009; Blonigen and Piger 2014), and what the net benefits are. The empirical literature is, however, far from arriving at a unanimous opinion on the net effects of FDI. In fact, the extent to which FDI is enhancing growth depends on the degree of complementarity and substitution between foreign and domestic investment (De Mello 1999; Munemo 2014). On the one hand, FDI has a complementary effect when the surge in foreign capital is associated with positive technological spillovers that increase the productivity of local enterprises and stimulate domestic investment and new local entry. Studies have shown that the extent to which an economy can reap the benefits from these knowledge externalities depends on its available stock of human capital (Borensztein et al. 1998; van Pottelsberghe and Porterie 2001). On the other hand, FDI has a substitution effect when the foreign investment crowds out equal amounts of investment from domestic sources by competing in product or financial markets. The increased competitive pressure might lead to the exit of local businesses and to a slow replacement of new local entry.

Most of the relevant literature looks at the impact of FDI on proxies of economic growth such as capital accumulation, total factor productivity (TFP) growth, and gross domestic product (GDP) growth. More recently, an increasing number of studies deals with the relationship between FDI (cross-border mergers and acquisitions, M&A) and new local firm’s entry or firm’s survival (De Backer and Sleuwaegen 2003; Ayyagari and Kosová 2010; Munemo 2014; Danakol et al. 2017). The generation of new businesses—or entrepreneurship in general—offers a new perspective to look at the effects of FDI on the host economy. The entry of new domestic firms is often seen as a key driver of economic growth and job creation, and it has become a primary goal for policy makers. As with the relationship between FDI and other measures of growth, the interaction between FDI and entrepreneurship is shaped by complex dynamics such as vertical and horizontal industry spillovers (Markusen and Venables 1999) and business start-up regulations (Munemo 2014). Consequently, the empirical literature provides ambiguous predictions about the relationship between FDI and entrepreneurship.

In addition, FDI inflows have different impacts on the host countries depending on the types of FDI such as greenfield FDI^1^ (new foreign firm) or cross-border M&A (foreign acquisition of an existing domestic firm). The traditional view on the impacts of FDI suggests that greenfield FDI is expected to increase the productivity, employment and capital formation of host countries, while cross-border M&A only involves a change from local to foreign ownership of existing assets and production capacity (Norbäck and Persson 2005; Johnson et al. 2006; Ashraf et al. 2016). Moreover, quite a few cases of cross-border M&As have been criticized as speculative funds seeking only the arbitrage profits with no value-adding contribution such as technology transfer or new investment for technological innovation (Kim 2009).

**Fig. 1:**
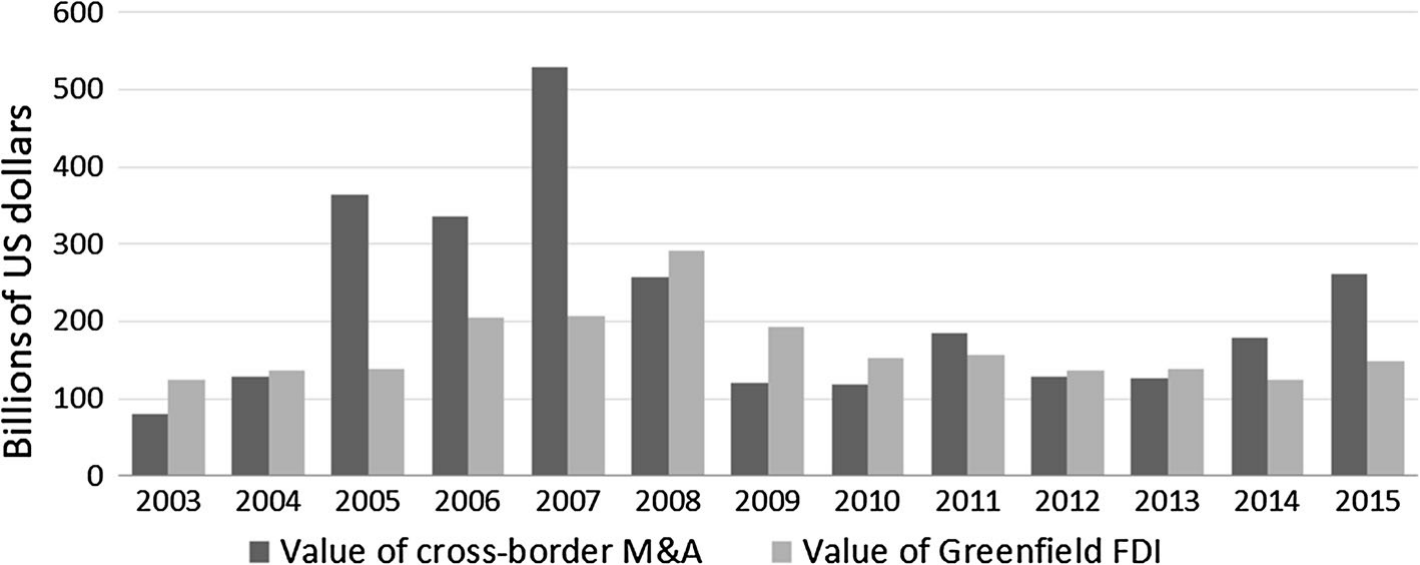
Values of cross-border M&A and announced greenfield projects in Europe. *Source* UNCTAD—Annex Tables.

Due to the lack of reliable data on greenfield FDI, the contribution of this type of investment to economic growth has been underinvestigated.^2^ However, greenfield FDI inflows in Europe account for more than 40% of total FDI. Figure 1 reports the values of both cross-border M&A and greenfield investment projects inflows in Europe. The figure shows both the pre- and post-crisis fluctuations and the recent slow growth recovery during 2014–2015. During the entire period 2003–2015, greenfield FDI and cross-border M&A amounted to USD 2.2 and 2.8 trillion, respectively.

In this paper, we use a novel data set on greenfield FDI from the fDi Market (www.fDimarkets.com) online database^3^ that allows us to investigate the less explored (short-term) effects of greenfield FDI on a specific dimension of the host economy, i.e. the entry of new domestic firms. We constructed a unique sector/country-level panel data set by matching information on greenfield FDI projects to domestic business birth rates from Eurostat and additional data on sector and country control variables from the Organization for Economic Cooperation and Development (OECD).

Previous empirical findings could be viewed as inconclusive because they typically rely on overall FDI inflows or only cross-border M&A. Our analysis complements the literature on the entry effects of inward FDI, as we are the first to evaluate the effects of greenfield investment on new domestic entry. Moreover, while the impact of FDI on firms and host economies has been largely studied, knowledge about the impact of knowledge intensive investment is limited. Although knowledge intensive activities such as R&D are still highly concentrated in headquarters, evidence shows that knowledge intensive FDI has grown substantially and more rapidly than the other types of FDI in the past decade (OECD 2008, pp. 219–248; Hall et al. 2011). In contrast with general FDI, evidence on the impact of foreign R&D stocks and the presence of foreign-owned high-tech firms points to a potential unambiguous positive influence on the domestic economy (Coe et al. 2009; Keller and Yeaple 2009). Indeed, more than other types of FDI, investments such as design and R&D seem to favour the formation of specialized clusters and allow host locations to integrate in more competitive global value chains (Cantwell and Piscitello 2000; Carlsson 2006). For this reason, we narrow our focus on the effects of knowledge intensive greenfield FDI and investigate whether the ‘most desirable’ type of FDI (greenfield and knowledge intensive) is indeed a transmission channel of knowledge and entrepreneurial skills or if, as with other types of foreign investment, there are the opposing effects on the creation of domestic businesses found in the literature.

The main contribution of our paper consists in adding a new piece of evidence to the FDI-entrepreneurship puzzle which may be extremely relevant from a policy perspective. Indeed, national governments have adopted several competitive strategies to attract greenfield FDI, and in particular knowledge intensive investment. Blomström et al. (2003) observe that policymakers mainly compete for greenfield FDI, by means of subsidies and incentives, as voters seem to reward politicians for attracting new investment projects (UNCTAD 2000).

The remainder of the paper is organized as follows. The next section presents a brief review of the literature. Section 3 describes the data and the methodological approach. Section 4 discusses the results of the econometric estimations. Section 5 concludes.

## Domestic entry and the role of FDI

The generally held assumption in the theoretical literature is that foreign firms not only bring new investments that creates new jobs and boosts national income, but also spill over some of their knowledge to local firms that are, as a consequence, able to increase their productivity. The potential to increase productivity is conditional on structural characteristics of the host economy, such as the absorptive capacity and the technological distance to the source country of the FDI (Görg and Greenaway 2004; Görg and Strobl 2005). Markusen and Venables (1999) and Rodríguez-Clare (1996) explain that the potentially favourable effects of FDI on the host country are the consequence of the generation of “forward” and “backward linkages”^4^ (vertical and horizontal spillovers). Even though a new FDI project creates additional competition in a local industry, the possible consequent reduction in prices may be beneficial to customer firms. Rodríguez-Clare (1996) shows that FDI can lead to the development of a local industry which may become sufficiently competitive to eventually drive the MNEs out of the market. Also, if the costs of communication between the headquarters and the production plants are high, the entry of foreign firms can increase the demand for local specialized input suppliers. Grossman (1984), on the other hand, indicates that an inflow of foreign enterprises has a negative effect on the supply of local entrepreneurs. In fact, the local entrepreneur is faced with the choice between the entrepreneurial income and the wage employment at a multinational firm, which typically has a higher wage premium to retain the employees and avoid knowledge spillovers (Glass and Saggi 2002).

While the theoretical literature tends to be more cohesive, the empirical analyses of the FDI spillovers on host economies have offered mixed results at best. In a review of 40 empirical studies, Görg and Greenaway (2004) reported that only eight studies employing panel data find an unambiguous positive aggregate impact of FDI, and only for developed economies.

Although earlier studies on the impact of FDI on domestic investment found a positive impact for both OECD countries (De Mello 1999) and developing countries (Borensztein et al. 1998), more recent work reports mixed evidence of no or negative effects on domestic investments (Agosin and Machado 2005). More precisely, Wang (2010) finds that there are negative contemporaneous effects on the ratio of gross fixed capital formation to GDP, especially among developed economies, whereas he finds positive cumulative effects for developing countries. Munemo (2014) investigates the impact of FDI and entry regulation on domestic investment and shows that FDI crowds out domestic investment if the entry costs are too high.

Other studies have investigated the impact of foreign investment on local firms’ productivity using firm-level data. Results point, in general, to the effects of negative spillovers on the domestic firms for transition economies (Konings 2001; Javorcik and Spatareanu 2008; Djankov and Hoekman 2000),^5^ Southern European economies (Barbosa and Eiriz 2009; García et al. 2013), and developing economies (Aitken and Harrison 1999). However, the negative firm-level effect disregards the fact that technology transfer is a process that requires time and learning resources. Liu (2008) finds that an increase in FDI lowers the short-term productivity level but raises the long-term productivity growth rate of domestic Chinese firms in the same industry. Furthermore, the effect of FDI may differ between firms. As Iacovone et al. (2015) show for the case of Walmart entering the Mexican market that firms in the lowest quartile of the size distribution are affected negatively, while larger local firms may benefit from FDI.

The literature on firm entry/exit and occupational choice also deals with the impact of FDI on domestic economies, and it points to both positive and negative effects of the foreign presence on local economies. Extending Jovanovic’s 1994 model of firm formation to allow for entry of foreign firms, the study of De Backer and Sleuwaegen (2003) predicts that the foreign firms crowd out local ventures. They argue that since MNEs pay higher wages and skim the labour market, the stronger rise in wages than in entrepreneurial income stimulates people to become workers instead of entrepreneurs. However, their empirical results also suggest that “the importance of positive long-term structural effects—learning, demonstration, networking and linkage effects—between foreign and domestic firms can moderate or even reverse crowding out effects” (De Backer and Sleuwaegen 2003, pp. 16–17). Similar results are found in the study of Zajc Kejžar (2011) who tests the effects of greenfield FDI and cross-border M&A and finds that only greenfield FDI decrease the survival probability of Slovenian firms. However, the crowding-out effect diminishes as the export propensity of local firms increases, while the presence of foreign affiliates reduces the exit probability of their downstream local customers, via positive forward linkages. Ferragina and Mazzotta (2014) investigate the effects of forward and backward linkages between foreign and Italian firms and find no clear evidence of an impact of foreign presence on the exit of local firms. They do, however, find that FDI reduces the probability of exit of highly productive firms or of firms in medium and low-tech sectors. These results may be explained by the differences in absorptive capacity.

The study of Bürke et al. (2008) develops an additional conceptual framework to explain the contrasting positive industrial spillovers and the crowding out effects. They hypothesize that the impact of FDI varies between dynamic and static industries. Dynamic markets, typically characterized by a high rate of firm churn, are more innovative and competitive. In these markets, foreign firms are more likely to displace domestic firms, as foreign companies are more innovative and have more competitive technologies. In contrast, static markets are associated with later stages of innovation diffusion. In static industries domestic firms are more imitative and have more scope to benefit from the foreign technology spillovers.

More recently, a growing number of studies have recognized the importance of exploring the relationship between FDI and entrepreneurship, as this may drive economic growth (Acs 2006; Fritsch and Wyrwich 2017). According to the knowledge spillover theory of entrepreneurship (Acs et al. 2009, 2013), the creation of new firms also provides a ‘conduit’ with which knowledge brought into the country by FDI is transferred to the local economy. By comparing Ireland and Wales, (Acs et al. 2012) hypothesise that, depending on the type of FDI and the accompanying local entrepreneurship policies, FDI may increase knowledge-based entrepreneurship in a country.

On the one hand, studies that have focused on one single country find evidence of positive spillovers from FDI on domestic entrepreneurship [Liu et al. (2014), Anwar and Sun (2015) for China; Ayyagari and Kosová (2010) for the Czech Republic; Barrios et al. (2005) for Ireland]. Similar to Barrios et al. (2005), Lee et al. (2014) show that the positive effect only holds up to a certain degree of FDI intensity in a region. Beyond a certain threshold, local entrepreneurship tends to diminish because of too high competition and entry costs and a comparably high attractiveness of foreign firms as workplaces for potential entrepreneurs.

On the other hand, results from cross-country/industry analyses point to the opposite direction. In particular, Albulescu and Tămăşilă (2016) does not find any association between inward or outward FDI on entrepreneurial activity in 16 European countries. Colantone and Sleuwaegen (2010), using data on eight countries from the “Business Demography Statistics” database by Eurostat, find a strong displacement exit and a slow replacement entry due to trade exposure. Pathak et al. (2015) analyse cross-country Global Entrepreneurship Monitor (GEM) survey data on entrepreneurs from 38 countries and find that inbound FDI is negatively associated with five types of entrepreneurship (nascent, new, early-stage, established, and high-growth). Similarly, Danakol et al. (2017), find a negative impact of M&A on three different types of entrepreneurship, controlling for corruption and cultural characteristics.

The few existing cross-country studies suggest a neutral or negative relation between inward FDI and domestic entrepreneurship; however, we still do not know whether we could generalize and conclude that all FDI inflows have the same impact on the formation of new local businesses.

To address this issue, our study contributes to this latter emerging literature with the analysis of the role of a specific type of FDI, namely the greenfield knowledge intensive FDI. Greenfield FDI, differently from cross-border M&A, is considered to increase the productivity of domestic firms, and knowledge intensive FDI activities, or FDI activities in knowledge intensive sectors (Antonietti et al. 2015), are regarded as the investment with the highest potential for knowledge spillovers for both international investors and local competitors, universities and research institutes (Castellani and Pieri 2016; Castellani et al. 2016).

## Data and descriptive statistics

This section describes our dataset (Sect. 3.1), the variables used (Sect. 3.2) and descriptive statistics (Sect. 3.3).

### Dataset construction

For our empirical analysis, we created a data set from three data sources which allow us to analyse the impact of knowledge intensive greenfield FDI on business entry in the host country for the first time: the fDI Markets database of the Financial Times, the Structural Business Statistics (SBS) of Eurostat, and industry level information from OECD databases.

The fDi Markets is an on-line database maintained by fDi Intelligence, a division of the Financial Times Ltd. Since 2003, fDi Intelligence collects available information from company data and media sources on greenfield foreign direct investments and monitors cross-border investments covering all sectors and countries worldwide. Data from the fDi Markets database are used by the UNCTAD to present global investment trends in the World Investment Reports series^6^ and have been used in publications by the Economist Intelligence Unit and in recent academic research (e.g. Crescenzi et al. 2014; Paniagua and Sapena 2014; Castellani and Pieri 2016; Castellani et al. 2016; Amoroso et al. 2015; Antonietti et al. 2015). The data are provided at the project level and includes information on the project date (the month when the FDI project started), the name of the investing company, the source and destination addresses of investment at the city level, the estimated amount of capital invested, the industry sector in which the investment takes place, and the type of activity. The latter covers five different classes of knowledge intensive activities which are design, development, and testing; education and training; headquarters activities^7^; information and communication technologies and Internet infrastructure; and research and development.

Our second data source is the structural business statistics (SBS). The SBS is a database on firm dynamics maintained by Eurostat, which covers, among others, the number of firm births, the number of firm closures and the number of active enterprises in the EU member states. The data are available for the EU-28 countries since their respective entry into the EU. As with all other statistics from Eurostat, the data for the SBS are assembled by statistical agencies of member states and originate in the national business registers. The national statistical offices collect the data based on internationally harmonised rules for data collection and preparation. The data are available at the sector level. Based on this information firms can be classified into different subgroups according to their sector affiliation.

In order to control for factors that are found to be relevant for the formation of new firms at the sector level we make use of two databases from the OECD. The first is the OECD Main Science and Technology Indicators database (OECD-MSTI) which provides information on the activities of the OECD countries in the field of science and technology such as R&D expenditures. The second is the database for Structural Analysis (STAN) which includes measures of output and inputs at the industry level. From this database, we extract the value added, the gross operating surplus and mixed income, the gross capital stock, the labour costs, the number of employees and the production value.

Our data sample covers the period between 2005 and 2012. The focus on this period is mainly due to the availability of the relevant data at the time of the analysis. Because we lag our independent variables by one year in order to avoid simultaneity bias, the data on the cross-border greenfield projects from the fDi Markets database as well as the aggregate production and R&D statistics from the OECD cover the period 2004–2011.

Matching and integrating heterogeneous data sources allows us to analyse the impact of knowledge intensive greenfield FDI on the local entrepreneurship; it presents, however, several challenges. One above all is the very small matched sample size (45–88 observations) deriving from the different patterns of missing observations across the various data sets. To mitigate this problem, we decided to impute the missing values of the total number of active firms in the SBS^8^ (needed to compute both foreign and domestic entry rates). To impute the number of total active firms, we assume that the stock of firms has grown at a constant rate between 2004 and 2012. We calculate the average growth rate with the available data points, and then extrapolate the time series backwards. We checked whether this data manipulation affects our results by comparing the outcomes of our estimations with and without the imputed values. We do not detect any qualitative differences.^9^ For the analyses, we consider all the observations for which data on the full set of variables (dependent and independent) is available. The resulting sample of 454 observations covers 10 EU countries and 20 industries.

### Variables specification

Table 1 shows the variables used in the analysis including their data sources. Our dependent variable is what we call the ’domestic entry rate’ which is defined as the number of births of enterprises relative to the number of active enterprises in a given year and sector (Ayyagari and Kosová 2010; Kosova 2010). SBS data on enterprise births refer to the birth of firms that are not foreign-controlled.^10^ Dividing the number of births by the number of active firms makes the number of firm births comparable between economies of different size.

**Table 1:** Variable description

Variable name	Description	Source
Domestic entry rate (*DomEntry*)	The ratio between the number of births of enterprises and the total number of active enterprises at time *t* in 20 business sectors (except activities of holding companies) and in 10 countries. Data are collected for the reference period 2004–2012	Structural business statistics—Eurostat
Foreign entry rate (*ForEntry*)	The ratio between the number of knowledge intensive greenfield projects and the total number of active enterprises at time *t*. Knowledge intensive greenfield FDI are defined as cross-border greenfield investment projects in R&D, design, development and testing, education and training, headquarters activities$^{\mathrm{a}}$, and information and communication technologies. The data are at project level, however we aggregate the total number of projects per sector and year, as the other data sources are aggregated at sector- or country-level	*fDi Markets* database
*Dyn*	A dummy with value of one if firms churn rate—sum of domestic entries and exits, divided by the total number of active firms—is larger than 15%, zero otherwise	Structural business statistics—Eurostat
*Tech*	A dummy with value of one if the NACE sector is a medium/high-tech sector, zero if it is a low-tech sector. (For the division of the sectors into medium/high-tech and low-tech sectors see next Table 2)	”
Price-cost margin (*pcm*)	An approximation of the price-cost margin, $\frac{p-c}{p}$, is given by multiplying and diving by the demanded quantity *q* to obtain profits over sales. For this calculation, we divide the gross operating surplus and mixed income by the production value	Authors’ calculations based on the variables provided in the OECD STAN database
Industrial gross domestic product growth ($\varDelta gdp$)	Growth rate of the gross production per sector	”
Capital intensity (*CapInt*)	The capital to labour ratio, given by the gross capital stock divided by the labour costs	”

$^{\mathrm{a}}$ Headquarter activities in MNEs are high-skill activities such as R&D, marketing and management (Bandick et al. 2014). Although the decision to open a headquarter abroad is mainly driven by low corporate taxes, HQs are found to be located in areas with similar industry specialization and with high levels of business services, which are typically knowledge intensive (Falk 2012)

Our focal right-hand-side variable is the foreign entry rate; it is defined similarly to other studies (De Backer and Sleuwaegen 2003; Colantone and Sleuwaegen 2010; Zajc Kejžar 2011) as ratio between the number of knowledge intensive greenfield FDI projects from the fDi Markets database and the number of active firms in the host country, for each year.

We include a set of control variables that have been shown to be relevant for the formation of new firms at the firm level. In particular, we control for the R&D intensity of the sectors. In particular, we group sectors into low-tech and medium/high-tech sectors to analyse whether the potential knowledge spillovers differ between the levels of R&D of sectors. For this grouping we applied a classification from the OECD (see Table 2). R&D provides a source of new ideas that entrepreneurial businesses can transform into new products—i.e. R&D opens up new opportunities. Wennekers et al. (2002) report evidence that technology change is one of the main reasons for expanded entrepreneurial opportunities. In addition, Audretsch et al. (2008) show that a high regional R&D activity and proximity to research institutions such as universities increase the opportunities to start new knowledge-based businesses.

**Table 2:** Industry grouping

ISIC	Sector name	ISIC	Sector name
*Low-tech sectors*		*Medium/high-tech sectors*	
10t12	Food products, beverages and tobacco	05t09	Mining and quarrying
13t15	Textiles, wearing apparel, leather	19t22	Manufacture of coke and refined petroleum products; chemicals and chemical products; basic pharmaceutical products and pharmaceutical preparations; rubber and plastic products
16	Wood/wood products (except furniture)	23	Manufacture of other non-metallic mineral products
17t18	Paper and printing	24t25	Manufacture of basic metals and fabricated metal products, except machinery and equipment
45t47	Wholesale and retail	26t28	Manufacture of machinery and equipment n.e.c.
55t56	Hotels and restaurants	29t30	Manufacture of motor vehicles, trailers, semi-trailers and of other transport equipment
		31t32	Manufacture of furniture; other manufacturing
		35t39	Electricity, gas, steam and air conditioning supply; Water supply; sewerage, waste management and remediation activities
		41t43	Construction
		49t53	Transportation and storage
		58t63	Information and communication
		64t66	Financial and insurance activities except activities of holding companies
		68t82	Administrative and support service activities
		84t99	Education; human health and social work activities; arts, entertainment and recreation; other service activities

Moreover, researchers studying domestic entry and exit argue that firm entry is the response to profitable opportunities that depend on price-cost margins (profitability) and on the economic growth of the domestic industry (Hause and Du Rietz 1984; Shapiro and Khemani 1987; Geroski 1989; De Backer and Sleuwaegen 2003). Past profitability signals profitable opportunities to domestic entrepreneurs and a rapid growth of the sectoral GDP of the domestic market indicates a large market potential which leads to a high entry rate of firms. The sectoral growth rate also allows us to control for other sector specific cyclical effects which may impact entry. We therefore include profit over sales as an approximation of the price-cost margin and the GDP growth at the sectoral level in the regression.

Theoretical and empirical models have shown that entry into a market can be deterred in the presence of high entry costs in the form of capital requirements (Khemani and Shapiro 1986). To capture this effect we control for the capital intensity of an industry in the regressions.

In addition, we follow the idea of Bürke et al. (2008) and analyse differences between dynamic and static industries. For this, we group the industries by their firm churn rate. The churn rate is commonly defined as the number of firm entries plus exits relative to the stock of firms. We calculate the firm churn rate using the number of firm births, firm deaths and active firms provided by Eurostat and define an industry to be dynamic (static) if the firm churn rate is above (below) the threshold value of 15% across all industries.

Overall, we expect the coefficients of our control variables to be consistent with the findings of the literature, i.e. new firms enter in rapidly growing industries, with relatively high price-cost margins and low entry costs, and where there is a relatively high level of technological opportunity or R&D intensity (Acs and Audretsch 1989).

### Descriptive statistics

Table 3 shows summary statistics of our sample. The average domestic firm birth rate is 7.12%, while the number of knowledge intensive greenfield foreign projects is 0.02%. Although having a comparatively low average value, the foreign entry rate is among the variables with the highest dispersion. The coefficient of variation (SD/mean) of this variable is 4 which makes it the second most dispersed variable. The variable with the highest dispersion is GDP growth (coefficient of variation 17.12). The coefficient of variation of the other variables ranges between 0.52 (firm churn rate) and 1.19 (capital intensity).

**Table 3:** Summary statistics

	Mean	Median	SD	Min	Max	N
*DomEntry*	7.12	6.01	4.48	1.43	42.34	454
*ForEntry*	0.02	0.00	0.08	0.00	1.06	454
*Tech*	0.72	1.00	0.45	0	1	454
*Churn*	13.38	12.02	6.95	2.51	59.48	454
*Dyn*	0.34	0	0.48	0	1	454
*CapInt*	10.38	5.58	12.34	0.98	71.76	304
$\varDelta gdp$	0.79	0.94	13.50	−36.46	82.93	370

**Fig. 2:**
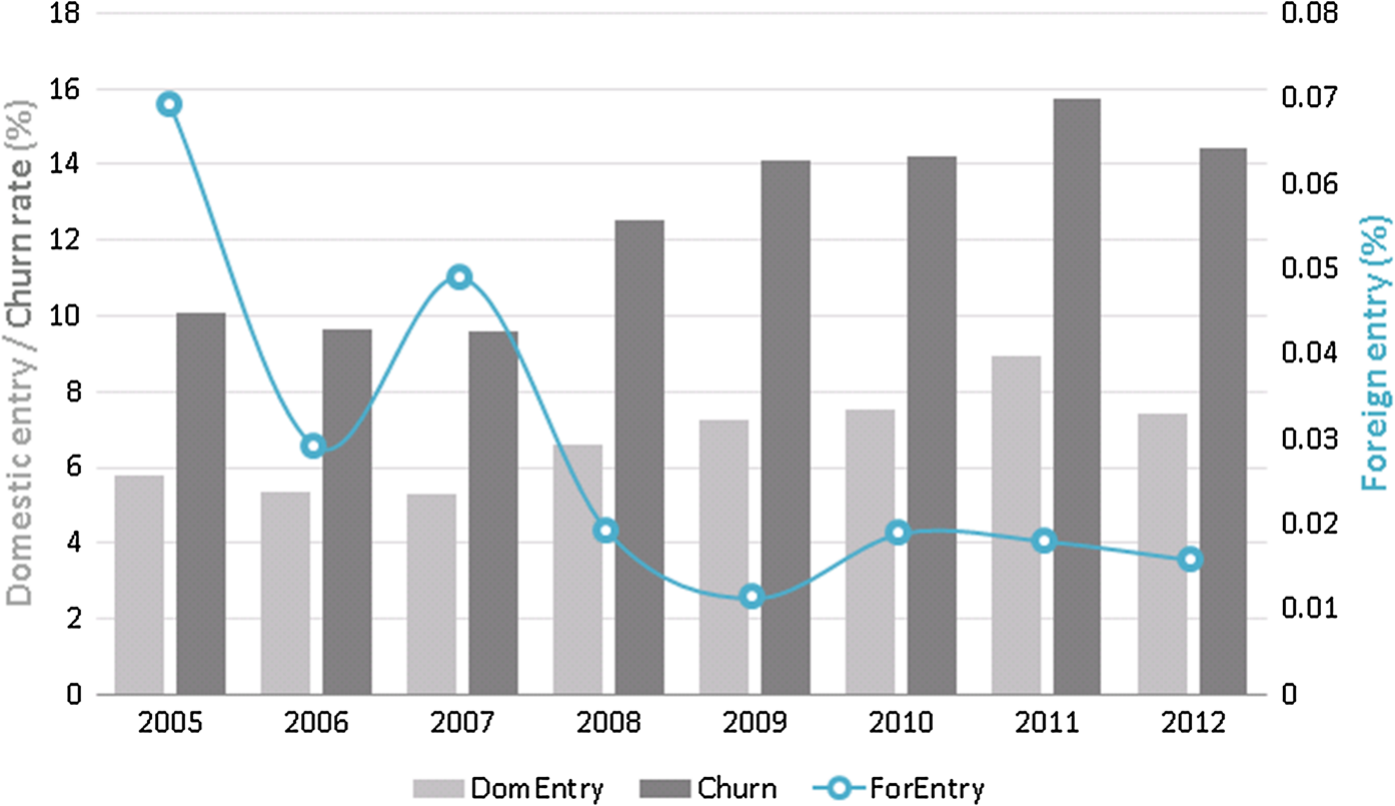
Trend over time of domestic entry rate, firm turnover and foreign entry rate (yearly averages, %). *Source* Own calculations based on *fDi Market* data

Figure 2 reports the averages over years and sectors of domestic and foreign entry rates as well as firm churn rate. The period of the European financial crisis 2007/2008 visually appears to have changed the trend in the series of all three variables. Both domestic entry and firm churn rate increased noticeably after these years. In contrast, the entry rate of new knowledge intensive foreign firms significantly went down in the years after the crisis.

Table 4 reports the averages over years and sectors of domestic and foreign entry rates, as well as the sectoral churn rate by country. There is observed variation among the countries. The domestic entry rate varies from 3.92% in Belgium to 15.04% in Estonia. Austria, Belgium, and the Netherlands have the highest inflows of knowledge intensive greenfield FDI projects (4 per 10 thousand active firm in the period of observation). The firm churn rate varies from 6.49% for Belgium to 27.84% for Estonia.

**Table 4:** Domestic, foreign entry rates, and firm turnover by country (averages, %)

Country	*DomEntry*	*ForEntry*	*Churn*
Austria	5.35	0.04	9.66
Belgium	3.92	0.04	6.49
Czech Republic	8.75	0.01	16.26
Denmark	9.56	0.01	19.84
Estonia	15.04	0.01	27.84
Finland	6.81	0.00	13.48
France	8.59	0.02	15.29
Germany	9.21	0.01	15.92
Italy	6.24	0.00	12.42
Netherlands	7.17	0.04	14.52

Table 5 displays and tests the difference in the means of the variables by R&D level and by firm churn rate. The domestic entry rates do not differ significantly between medium/high-tech and low-tech sectors. However, the foreign entry rates are higher in medium/high-tech sectors than in low-tech sectors. Also, as expected, the price-cost margin, the capital intensity, and the sectoral GDP growth are higher in the more technology intensive sectors.

Comparing static and dynamic sectors, domestic entry is higher in dynamic sectors than in static ones. In contrast, the foreign entry rate is lower in dynamic sectors. This is perhaps a consequence of the fact that multinational companies seem to be concentrated in countries with a lower firm churn rate (see Table 4). There is no significant difference in the price-cost margin, capital intensity, and GDP growth between static and dynamic sectors.

**Table 5:** Means of variables by sector type

Sector	Low-tech	Medium/high-tech	Static	Dynamic
*DomEntry*	6.55	7.35	4.88	11.41*
*ForEntry*	0.00	0.03*	0.03	0.01*
*pcm*	15.64	23.86*	20.36	24.11
*CapInt*	5.72	12.22*	9.71	11.74
$\varDelta gdp$	−2.41	2.09*	0.70	0.97

* Mean-difference test significant at 1%-level. The classification of the industries into low-tech and medium/high-tech can be taken from Table 2 in the Appendix. Static (dynamic) industries are those with a firm churn rate below (above) the median churn rate in the sample

Table 6 displays the correlation coefficients for all the variables used in the empirical specification. Domestic entry is negatively correlated with foreign entry rates and (highly) positively correlated with firm turnover, profitability (price-cost margin), and capital intensity. Foreign entry in knowledge intensive activities is positively correlated with higher level of technological capabilities, and negatively correlated with the level of firm churn rate, which formally supports the observation from Table 4 that knowledge intensive FDI projects tend to be carried out in countries with low firm churn.

**Table 6:** Correlation table

Variables	(1)	(2)	(3)	(4)	(5)	(6)	(7)
(1) *DomEntry*	1.00						
(2) *ForEntry*	−0.13*	1.00					
(3) *Dyn*	0.69*	−0.13*	1.00				
(4) *Tech*	0.08	0.14*	−0.04	1.00			
(5) *Pcm*	0.24*	−0.13	0.11	0.24*	1.00		
(6) *CapInt*	0.20*	−0.11	0.08	0.24*	0.73*	1.00	
(7) $\varDelta gdp$	0.05	0.03	0.01	0.15*	0.13	0.12	1.00

* Correlation coefficient significant at 1%-level

## Econometric analysis and results

### Empirical model

To investigate the impact of greenfield foreign entry on domestic entry, we regress the domestic entry rate of sector *j* of country *i* at time *t* on the past value of domestic entry rate, knowledge intensive greenfield foreign entry rate and a set of control variables, which have been identified by the literature as key determinants of entry. The empirical model is as follows

1 $$\begin{aligned} DomEntry^i_{jt}=\alpha + \beta DomEntry^i_{jt-1}+\gamma ForEntry^i_{jt-1} +\delta ^{\prime }X^i_{jt-1}+\epsilon ^i_{jt}, \end{aligned}$$

where *X* is a vector of lagged control variables at sector-level, including the growth rate of gross domestic product ($\varDelta gdp$), profitability (*pcm*), capital intensity (*CapInt*), the firms churn rate dummy (*Dyn*), and the level of technology (*Tech*). $\epsilon _{ijt}$ is a composite error term which includes year, country and sector fixed effects, and a remainder error assumed to follow an iid normal distribution. The coefficient $\beta$ measures the impact of greenfield foreign entry rate (or the relative foreign entry rate) on the domestic entry rate.

Given the ambiguous results found in the literature concerning the sign of the impact of foreign entry, we test whether this coefficient vary by sector classification. In particular, following some of the predictions in the literature, we interact the greenfield foreign entry variable with:

- The churn rate dummy, *Dyn*, a dummy taking value of one if the sector has a churn rate above 15% (dynamic sector), and zero otherwise (static sector)
- The technological classification of the sector, *Tech*, a dummy taking value of 1 if the sector is a medium/high-tech one, zero otherwise

### Estimation technique

Our data set is an unbalanced panel with observations clustered by country, sector and year. Our regression analysis is based on generalized least square (GLS) accounting for panel heteroskedasticity (the variance varies for each country-sector pair). All control variables are lagged by one year to potentially correct for issues of reverse causality. Nonetheless, the endogeneity deriving from the characteristics of the host market that affect the choice of FDI location and type of investment may yield inconsistent results. Figure 2 reports both new domestic and new foreign greenfield entry rates, and the firm churn rate. While the churn rate and the domestic entry rate follow a similar increasing trend over time, the greenfield investments seem to have dramatically dropped after the crisis. However, there could be unobserved confounders (omitted variables) that are correlated with both the domestic entry rate and the foreign entry rate (e.g. government regulations, labour market arrangements, macroeconomic stability, etc.).

We account for these potential endogeneity biases and rely on the instrumental variable approach. Namely, we use a 2-stage least squares (2SLS) estimator. In line with Danakol et al. (2017), we use the weighted average geographical distance^11^ between source and host country as instrument, as this is a priori not linked to the domestic entry rate, while it largely influences the location decisions of foreign affiliates (Carr et al. 2001). In the first stage the foreign entry rate is regressed on the weighted distance, country, sector and year fixed effects, while in the second stage, we estimate Eq. (1) where we replace the lagged foreign entry rate with the predicted values of the first stage.

### Estimation results

Table 7 presents the estimation results of Eq. (1). The first column (1) reports the estimated regression coefficients of a simple specification where the domestic entry depends only on past greenfield foreign entry rate and country, sector, and year dummies. The coefficient associated with past foreign investment is positive and significant. A one percentage point increase in the foreign entry rate corresponds to a 3 percent increase in the domestic entry rate.

**Table 7:** Results from a feasible generalized least squares regression of $DomEntry^i_{jt}=\alpha + \beta DomEntry^i_{jt-1}+\gamma ForEntry^i_{jt-1} +\delta ^{\prime }X^i_{jt-1}+\epsilon ^i_{jt}$

*DomEntry*	Dep. var				
	(1)	(2)	(3)	(4)	(5)
*DomEntryLag*		0.209***	0.260***	0.207***	0.205***
		(0.020)	(0.016)	(0.019)	(0.019)
*ForEntry*	3.049***	−0.236	0.768	−14.374***	−16.328***
	(0.875)	(0.683)	(0.484)	(4.011)	(3.525)
*Dyn*		4.102***	4.086***	4.118***	4.025***
		(0.164)	(0.159)	(0.162)	(0.167)
*Tech*		−0.164	0.328***	−0.182	−0.170
		(0.264)	(0.079)	(0.260)	(0.260)
*CapInt*		−0.062***	0.008	−0.062***	−0.075***
		(0.009)	(0.007)	(0.009)	(0.012)
*pcm*		0.039***	0.011*	0.038***	0.036***
		(0.007)	(0.006)	(0.007)	(0.007)
$\varDelta gdp$		0.018***	0.017***	0.018***	0.018***
		(0.005)	(0.005)	(0.005)	(0.005)
Constant	6.275***	3.482***	2.552***	3.501***	3.567***
	(0.411)	(0.277)	(0.161)	(0.272)	(0.277)
$Dyn\times ForEntry$			8.116*		6.546*
			(4.713)		(4.012)
$tech\times ForEntry$				14.355***	16.020***
				(4.048)	(3.562)
($ForEntry_{dydx}$)			4.824*	−3.022***	−1.907
			(2.588)	(1.380)	(2.890)
Observations	454	454	454	454	454
Country, Sector, and Time FE	$\checkmark$	$\checkmark$	$\checkmark$	$\checkmark$	$\checkmark$

$ForEntry_{dydx}$ is the marginal effect of *ForEntry* calculated at means of *Dyn* and *Tech*

Standard errors in parentheses

*** *p* < 0.01; ** *p* < 0.05; * *p* ≤ 0.1

Column 2 displays the regression coefficients from a full specification that takes into account the past domestic new entry rate, sectoral knowledge capabilities, physical capital intensity, and the profitability and the growth of sectors’ output. The marginal effect of past foreign investment in knowledge intensive activities on new domestic entry is not significant when taking these factors into consideration.

On the one hand, our results differ from those of Colantone and Sleuwaegen (2010) and De Backer and Sleuwaegen (2003) who find entry-discouraging effects due to increased trade exposure in manufacturing industries of eight European countries. On the other hand, results for the estimated coefficients of the control variables confirm the empirical and theoretical findings of the relevant literature.

The coefficient measuring the impact of industry capital intensity (a proxy for potential entry costs) on entry rate has the expected negative sign. In particular, while capital intensity affects positively the probability of firms’ survival (Doms et al. 1995; Bernard and Jensen 2002), the role of capital intensity as a barrier to entry is well recognized (Khemani and Shapiro 1986) and observed in the empirical literature (Scarpetta et al. 2002; De Backer and Sleuwaegen 2003; Feizpour and Moradi 2014). Anwar and Sun (2015) control for capital intensity at firm level rather than industry level and find contrasting results. Industry price-cost margin and gross product growth have a modest positive effect on domestic entry rate. A 10% increase in the lagged profit margin or past industry growth is associated with 0.4 or 0.2% increase in the entry rate, respectively.

In columns (3), (4) and (5) we include the interaction between knowledge intensive foreign entry and the firm churn rate dummy (3), the technological level of the sector (4), and both (5). When interacting the knowledge intensive foreign entry with the business churn dummy, we find that as foreign entry rate increases, the domestic entry rate of dynamic sectors increases 8% faster than that of static sectors. Higher industry turnover is typically associated with lower barriers to business. This, in turn, attracts both domestic and foreign businesses. Contrarily, static industries may be characterized by older incumbents and higher entry costs. In these industries, MNEs have larger capital to face the barriers to entry and would eventually discourage the entry of local businesses. Our results contrast the “surprising” positive effects in static industries found by Bürke et al. (2008, p. 403); however, their study takes into consideration the survival of new firms, while we look at the formation of new domestic businesses. Also, they rely on firm-level data for just one country, UK, and proxy the foreign presence using the share of employment by MNEs, without distinguishing between the knowledge intensity of their activities.

In column (4), we include the interaction of knowledge intensive foreign entry rate and technology intensity.^12^ In line with the predictions of Acs et al. (2013), we find that the impact of foreign entry on domestic entry depends on the level of technology intensity: new foreign entry in high- and medium-tech sectors yields a 14% faster increase in the domestic entry rate (compared to low-tech sectors). Indeed, higher technological capabilities increase the level of absorptive capacity and the pool of unexplored knowledge, which is then commercialized and transformed into economic knowledge (new products and services). Different from the specification in column (3), the marginal effect of the foreign entry rate is negative and statistically significant (−3%). Anwar and Sun (2015) do not find any evidence of such positive R&D-entry relationship, however their measure of technological intensity is at the firm-level. Although the theoretical argument of Audretsch et al. (2005) and Acs et al. (2013)—regional knowledge capacity attracts FDI and results in knowledge intensive entrepreneurship—could apply also to firm-level R&D, the pool of latent entrepreneurs may benefit from the sectoral level of R&D. According to Feldman (1999), sectoral R&D is a proxy for the quality of human capital in the form of scientific and technical expertise. In addition, the level of sectoral R&D intensity accounts for intra-sectoral knowledge spillovers which may contribute to create the right climate to enable potential entrepreneurs to spot the profit opportunity and start a business.

These results are also in line with those of Görg and Strobl (2005), who argue that firms in high-tech sectors have a greater absorptive capacity and are able to benefit from the foreign technology spillovers, but they contrast with the results of Ferragina and Mazzotta (2014) who find that firms in high-tech sectors do not benefit from horizontal FDI while in low- and medium-tech sectors they do. Indeed, the presence of multinationals in high-tech sectors may increase the competitive pressure for domestic firms causing a costly reallocation of economic resources within and across sectors.

Finally, in column (5), we consider both the interactions with the sectoral turnover and the technological intensity. The results confirm that the new foreign knowledge intensive entry corresponds to 6.5 and 16% faster new domestic entry rates in dynamic and high/medium-tech sectors, respectively. The marginal effect of greenfield FDI is non-statistically significant.

Table 8 reports the results from 2SLS estimations, which point to a lack of association between new foreign and domestic entry rates. Below the estimation results, we present the results from the first stage, where the variable *ForEntry* is regressed on the logarithm (and squared logarithm) of the weighted geographical distance between the investing origin countries and the destination country. We also report a set of test statistics to assess the goodness of fit (F-statistic and R$^2$), the endogeneity of the foreign entry rate, and the relevance of the instruments used (test of underidentification). Using the 2SLS approach, differently from Danakol et al. (2017), we do not find any significant effect of the greenfield foreign entry rate on the domestic entry rate. In addition, the hypothesis of endogeneity of the knowledge intensive gFDI is always rejected, making the feasible GLS our preferred estimator for this empirical model.

**Table 8:** Results from 2SLS estimation of $DomEntry^i_{jt}=\alpha + \beta DomEntry^i_{jt-1}+\gamma ForEntry^i_{jt-1} +\delta ^{\prime }X^i_{jt-1}+\epsilon ^i_{jt}$

*DomEntry*	Dep. var				
	(1)	(2)	(3)	(4)	(5)
*DomEntryLag*		0.558***	0.381***	0.388***	0.345***
		(0.080)	(0.104)	(0.112)	(0.108)
*ForEntry*	−10.017	−14.549	−4.763	−13.557	−275.150
	(15.181)	(15.808)	(27.345)	(36.891)	(235.085)
$ForEntry\times Dyn$				16.667	
				(39.326)	
$ForEntry\times Tech$					275.530
					(105.612)
*Dyn*			3.751***	3.616***	3.741***
			(0.607)	(0.626)	(0.733)
*Tech*			−1.261	−1.489	−0.150
			(1.629)	(1.890)	(0.784)
*CapInt*			−0.058*	−0.067*	−0.040
			(0.032)	(0.037)	(0.040)
*pcm*			0.039	0.027	0.047
			(0.030)	(0.044)	(0.034)
$\varDelta gdp$			0.019	0.025	0.022
			(0.029)	(0.033)	(0.026)
Constant	7.060***	3.029***	1.508	1.674**	2.823**
	(0.986)	(0.915)	(0.942)	(0.808)	(1.274)
*Results from the first-stage*					
$dist_{GEO}$	0.185*	0.185*	0.045	0.027	0.010
	(0.101)	(0.101)	(0.099)	(0.099)	(0.013)
$dist^2_{GEO}$	−0.012*	−0.012*	−0.003	−0.002	−0.001
	(0.007)	(0.007)	(0.007)	(0.007)	(0.001)
Observations	267	267	267	267	267
R-squared	0.264	0.451	0.677	0.597	0.221
Uncentered R2	0.792	0.845	0.909	0.886	0.780
Endogeneity *p* value	0.433	0.321	0.841	0.718	0.363
Underidentification *p* value	0.0176	0.0179	0.504	0.582	0.110
F-stat	0.000	0.000	0.000	0.000	0.000

Robust standard errors in parentheses

*** *p* < 0.01; ** *p* < 0.05; * *p* < 0.1

## Discussion and conclusions

Foreign direct investment (FDI) is an essential part of an international economic system and potentially a major catalyst for economic development. The catalytic potential arises from the fact that FDI is usually accompanied with a transfer of technology and knowledge from the country of origin to the host country. In particular, this applies to knowledge intensive FDI where R&D activities are outsourced to another country than the country of the headquarter.

Yet, the benefits from FDI do not accrue automatically and evenly across countries and sectors. The main reason is that most of the knowledge is not codified but tacit. Thus, some transformative mechanism is needed to ensure that the knowledge transfer actually takes place. One such mechanism is the start-up of new firms. Individuals might be inspired to new business ideas by the knowledge existing in foreign firms and set up a new firm thereby transferring the knowledge from the foreign firm to the local economy.

In this paper, we constructed a unique country/sector-level panel data set to analyse how knowledge intensive greenfield FDI affects domestic economy, looking at the impact on the generation of new local businesses. Our main contribution to the literature on FDI spillovers is that we explore the empirically underinvestigated relationship between greenfield FDI and domestic entry. Compared to previous studies that rely on the largely available information on M&A, we build our analyses on the less explored data on greenfield knowledge intensive FDI, which is considered to be the ‘best’ type of FDI with the highest potential of positive effects for the host country.

We find that the short-run impact of this type of FDI on domestic entrepreneurship is generally positive as one would expect from greenfield knowledge intensive investment, and that the benefits from this type foreign entry are larger in more dynamic and technologically intensive sectors, than in static and low-tech sectors. The observed positive effects for dynamic and high- and medium-tech sectors validate some of the hypotheses advanced by the literature on knowledge spillover theory of entrepreneurship (Audretsch et al. 2006; Acs et al. 2012), and resonate with similar empirical studies (Liu et al. 2014; Antonietti et al. 2015), as in these sectors knowledge may spill over faster.

The knowledge transfer process, however, does not exclude the possibility to observe some initial negative effects on the domestic entry rate, due to adjustments. Potential entrepreneurs have to first learn about new business opportunities resulting from the FDI project by engaging with this new source of knowledge.

Moreover, as Acs et al. (2013) argue, for knowledge spillovers to take place, linkages to the source of knowledge are necessary. This is due to the tacit character of most of the knowledge. Individuals have to enter into direct exchange with knowledge carriers to take up new insights. Two possible ways in which this can occur are if individuals from the domestic workforce become employees in the affiliates of the foreign firm or if they serve as business partners (Fu 2012). In both ways, the process of knowledge spillover takes time.

Although we are able to make an important step forward to analyse the effect of the most desirable type of FDI, our analysis has its limitations, due to the lack of appropriate data. First, we consider only the short-run effect of knowledge intensive greenfield FDI on the local start-up rate. Admittedly, knowledge spillovers from FDI to the local economy is a complex matter which is not fully captured by considering only one year lag in the regressions. However, testing the effects of greenfield FDI with a longer lag specification would result in a smaller sample size. Second, we only estimate the direct effect of FDI and do not consider indirect effects through backward and forward linkages, although these have been shown to be relevant in the literature. In fact, we only have quite rough information on the sector in which the FDI occurs (at most on the two-digit level). This implies that we cannot distinguish between vertical and horizontal spillovers, and therefore cannot map the linkage structure. Third, as discussed in Sect. 3, we imputed the data on the stock of active firms. We tested the importance of the bias from imputing the total number of active firms and we found it to be negligible. Fourth, because of the low number of full observations available, we use a parsimonious regression model. In particular, we only included the capital intensity to capture entry costs. There are, of course, other factors that influence domestic entry, such as red tape or institutional factors (e.g., corruption, the degree of enforcement of property rights, etc.). However, like other studies before ours, we assume these factors are captured by country and sector dummies.

Nevertheless, we think that some policy implications can be derived from our results. Based on the positive spillovers expected from greenfield or knowledge intensive foreign investment, national governments have adopted several competitive strategies to attract FDI, often racing to bottom of labor and fiscal standards (Javorcik 2004; Olney 2013). While technologies and knowledge may eventually spill over on the host economy, policy makers should also consider that, in the short run, these spillovers mainly concern the entrepreneurial activity in high-tech and dynamic sectors. In terms of industrial policy, this suggests that the focus on leading sectors—where both incumbent and younger entrepreneurial firms have representatives—requires an alignment of industrial and firm-level policies. New industrial policies (e.g., targeted policies in advanced manufacturing, green economy, etc.) can be seen as public sector interventions aimed at changing the distribution of resources across economic sectors, while firm-level policies play an important role in reallocating resources to market segments that might become more productive. In this regard, in addition to generic enterprise policies (e.g., strengthening innovation framework, improve access to risk and human capital, etc.), a specific policy for leading sectors that focuses on the interaction between national and local governments, research institutes, and young innovative enterprises, may increase the return to public funds to R&D.

Further research efforts should explore the long-run effects of knowledge intensive FDI, the role of country-specific institutions, and the type of entrepreneurship that is affected by foreign investment. Our future research agenda therefore includes the analysis of greenfield FDI and its impact on different types of start-ups, controlling for information on knowledge infrastructure at the regional level.

## Electronic supplementary material

Below is the link to the electronic supplementary material.

Supplementary material 1 (pdf 43 KB)

## Footnotes

1 A greenfield investment is the creation of a subsidiary from scratch by non-resident investors (www.imf.com).

2 See Ashraf et al. (2016) for a recent review.

3
*fDi Markets* is an online database maintained by fDi Intelligence, a division of the Financial Times Ltd.

4 A forward linkage is the indirect positive effect of FDI on a sector due to the increased competition in the sector where FDI takes place. The tougher competition in the sector with higher foreign presence may result in a reduction of prices and stimulate the demand of customer firms in other sectors. A backward linkage is the increased demand for local products and services and this backward effect may also strengthen the local supply industries (think of the ‘East-Asia effect’ on local suppliers).

5 In contrast, Javorcik (2004) finds positive FDI productivity spillovers via linkages with local suppliers in upstream sectors for Lithuanian firms.

6
http://unctad.org/en/pages/DIAE/World%20Investment%20Report/WIR-Series.aspx.

7 Headquarter activities in MNEs are high-skill activities such as R&D, marketing and management (Bandick et al. 2014). Although the decision to open a headquarter abroad is mainly driven by low corporate taxes, HQs are located in areas with similar industry specialization and with high levels of business services, which are typically knowledge intensive (Falk 2012).

8 Data from SBS is still under construction. The values for the number of active firms are absent for the earlier years of our period of observation. This, in turn, is mainly due to the notorious difficulty in determining the number of firm exits which are needed for the calculation of the stock of the firms.

9 The results of the comparison are available upon request from the authors.

10
http://ec.europa.eu/eurostat/statistics-explained/index.php/Structural_business_statistics_overview.

11 We use foreign capital expenditure to weight the geographical distance. Following a common practice in the literature, we take the great circle distance between nations’ capital cities where a sphere is used to approximate the shape of the earth.

12 The official classification adopted by the Eurostat http://ec.europa.eu/eurostat based on the average R&D intensities of the manufacturing and service sectors. So, for example, the sector of rubber and plastic products is defined as medium-low-tech for all years and all countries.
