# Supplementary material for: The short-run effects of knowledge intensive greenfield FDI on new domestic entry
Source: J Technol Transf. 2017 Apr 4;43(3):815–36. doi: 10.1007/s10961-017-9575-y (PMC6548471; doi:10.1007/s10961-017-9575-y)
Supplement: Supplementary file 1 — Supplementary material 1 (pdf 43 KB) [file 10961_2017_9575_MOESM1_ESM.pdf]

Appendix not intended for publication

The role of technological spillovers

In this Appendix, we investigate the presence of technological spillovers and their role in explaining the relationship between domestic and foreign entry. To identify technological and human capital spillovers, we consider two variables: the distance in high-tech exports (% of manufactured exports) and the distance in educational attainment (% of the population older than 25 with a completed tertiary cycle), respectively. The data comes from the World Development Indicators database (http://data.worldbank.org). One limitation when using these two measures of distance is, however, the number of observations. Indeed, the distances between investing and destination countries are only available for a small number of years and only in those pairs of sectors/countries where foreign countries have invested. When matching these variables with the other data sets by country, sector, and year, the resulting matched sample size is too small to perform a robust regression analysis.

To circumvent the issue, we propose an approach that rests on a strong and questionable assumption. The assumption is that the distances in high-tech exports and education between investing and destination countries are constant over time. This means that, even if during one specific year, sector j in country H had not received any knowledge intensive gFDI, we assume it did, and from the same countries.

The distances in high-tech exports and education were calculated as the weighted average Euclidean distances between the investing country and destination country. We use the shares of foreign capital expenditure to compute the weights. To make an example, if sector j of country H received a total of Euro x over the period 2003-2012 from a number of investing countries, say k, then the weighted Euclidean distance is computed as

$$d_{jH} = \sqrt{\sum_k \frac{x_{kjH}}{x_{jH}} (d_{jH} - d_k)^2}.$$

In this way, we obtain two measures of distance that are varying across destination country and sector, but that are constant over time.

Table 1: Summary statistics for the technological and human capital distance measures

	mean	median	sd	N
$dist_{TECH}$	9.4	9.1	4.9	265
$dist_{EDU}$	10	8.3	6.4	221

Table 1 reports mean, median, standard deviation, and the number of observations available for the two measures of distance. Despite the two distances have statistically not significant different means, educational distance is more dispersed than technological distance. The sample

size of the data set merged with these additional variables is twice smaller (between 221 and 265 observations).

Table 2 reports the regression results of eq. (**??**) including the effects of technological and educational distances. In the first column, while the effect of foreign entry is neutral, the two measures of distance are negatively related to the local entry of new firms. In the next columns, the full set of control variables is added. The technological distance is no longer associated with the domestic entry, while the coefficient relative to the educational distance continues to have a significant and negative sign. The interaction between technological distance and gFDI (column 3) has a negative sign, while the interaction with the distance in education (column 4) has a positive sign. The two interaction terms maintain the same signs even when they are both included (column 5).

These results suggest that the possibility of knowledge transfer via the spillovers from the local establishment of new foreign companies has different implications depending on the type of distance. The higher the international technological distance between two sectors, the lower the domestic entry rate. Therefore, in line with the findings of Jaffe (1986), spillovers are generated in "technological proximity". Viceversa, as the distance in education increases, foreign entry raises the domestic entry rate. This is counterintuitive as foreign advanced technologies are usually absorbed by human capital that received a similar education to that of the investing country. The economic interpretation of these results is however subject to the assumption we have made to construct the measures of distance.

Table 2: Additional estimations with technological and educational distances

Dep. var: *DomEntry*	(1)	(2)	(3)	(4)	(5)
$DomEntryLag$		0.319***	0.320***	0.310***	0.311***
		(0.026)	(0.026)	(0.026)	(0.026)
$ForEntry$	0.704	1.855***	3.301***	-3.986	-4.465
	(0.444)	(0.538)	(1.146)	(3.228)	(3.240)
$dist_{TECH}$	-0.159***	-0.009	-0.007	-0.015	-0.009
	(0.013)	(0.016)	(0.016)	(0.016)	(0.016)
$dist_{EDU}$	-0.044***	-0.042***	-0.040***	-0.041***	-0.045***
	(0.015)	(0.013)	(0.013)	(0.014)	(0.014)
$ForEntry \times dist_{TECH}$			-0.222*		-0.314**
			(0.119)		(0.139)
$ForEntry \times dist_{EDU}$				0.665*	1.006**
				(0.383)	(0.407)
Dyn		4.135***	4.146***	4.167***	4.180***
		(0.252)	(0.254)	(0.261)	(0.261)
$Tech$		0.186	0.236	0.118	0.150
		(0.145)	(0.146)	(0.154)	(0.153)
$CapInt$		-0.052***	-0.052***	-0.051***	-0.051***
		(0.017)	(0.017)	(0.017)	(0.017)
pcm		0.068***	0.067***	0.057***	0.057***
		(0.013)	(0.014)	(0.015)	(0.015)
Δgdp		-0.009	-0.009	-0.008	-0.011
		(0.008)	(0.008)	(0.008)	(0.008)
constant	8.738***	1.801***	1.819***	2.236***	2.248***
	(0.138)	(0.385)	(0.391)	(0.429)	(0.427)
Observations	221	221	221	221	221

Robust standard errors in parentheses

*** $p<0.01$, ** $p<0.05$, * $p<0.1$
